# Overexpression of ubiquitin carboxyl-terminal hydrolase 1 (UCHL1) in boys with cryptorchidism

**DOI:** 10.1371/journal.pone.0191806

**Published:** 2018-02-05

**Authors:** Dorota Toliczenko-Bernatowicz, Ewa Matuszczak, Marzena Tylicka, Beata Szymańska, Marta Komarowska, Ewa Gorodkiewicz, Wojciech Debek, Adam Hermanowicz

**Affiliations:** 1 Paediatric Surgery Department,Medical University of Bialystok, Bialystok, Poland; 2 Biophysics Department Medical University of Bialystok, Bialystok, Poland; 3 Electrochemistry Department, University of Bialystok, Bialystok, Poland; Universite Clermont Auvergne, FRANCE

## Abstract

**Background:**

The ubiquitin-proteasome system regulate p53, caspase and Bcl-2 family proteins, and is crucial for the degradation of the defective germ cells in testes. Purpose: to evaluate the concentration of ubiquitin carboxyl-terminal hydrolase 1 (UCHL1) in the blood plasma of boys with cryptorchidism and if there is any correlation with patient age.

**Methods:**

Patients—50 boys aged 1–4 years (median = 2,4y.) with unilateral cryptorchidism. Exclusion criteria were: previous human chorionic gonadotropin treatment, an abnormal karyotype, endocrine or immunological disorders or any long-term medication. The control group—50 healthy, age matched boys (aged 1–4 years, median = 2,1y.), admitted to the Pediatric Surgery Department for planned herniotomy. To investigate UCHL1 in blood plasma of boys with cryptorchidism, we used a novel technique Surface PLASMON RESONANCE Imaging (SPRI).

**Results:**

The median concentration of UCHL1 in the blood plasma of boys with cryptorchidism, was 5-folds higher than in boys with inguinal hernia, whose testicles were located in the scrotum. We also noticed statistically significant difference between UCHL1 levels in boys with cryptorchidism up to 2 years old, and above 2 years old. Older boys, whose testicles since birth were located in the inguinal pouch or in the abdominal cavity, had higher concentration of UCHL1 in their blood plasma, than boys from younger group. In the group of cryptorchid boys, we also found slightly lower concentrations of INSL3, without statistical significance and no correlation with UCHL1 levels.

**Conclusions:**

Uchl1 concentrations in the blood plasma of boys with cryptorchidism, may reflect the heat-induced apoptosis of germ cells. Higher UCHL1 concentrations in older boys with undescended testicles, probably express intensity of germ cell apoptosis, more extensive when testicles are subjected to heat-stress for longer period. Further analyses of UCHL1 may help to elucidate its role in mechanisms influencing spermatogenesis.

## Introduction

Testicles which did not descend to the scrotum, are at risk of disturbed spermatogenesis and testicular cancer, caused by elevated temperature in the abdominal cavity or in the inguinal pouch. According to some authors, early surgical orchidopexy does not change the risk of malignant transformation [2]. Contrary to that, the United Kingdom Testicular Cancer Study Group concluded that, orchidopexy before the age of 10 years, reduced the risk of testicular cancer [3]. Still, the surgical treatment of innate cryptorchidism is advocated in early infancy [1].

Spermatogenesis is a complex process, during which apoptosis controls the amount of germ cells and eliminates impaired germ cells [4]. According to recent studies, ubiquitin-proteasome system is crucial to the regulation of this process. The levels of ubiquitin are under control of ubiquitinating enzymes and deubiquitinating enzymes (DUBs)[5]. DUBs are divided into ubiquitin C-terminal hydrolases (UCHs) and ubiquitin specific proteases (UBPs) [5]. Kwon et al. suggested that one of those enzymes—ubiquitin Carboxyl-terminal hydrolase 1 (UCHL1) “plays a role in balancing the expression of apoptosis-inducing and apoptosis-protecting proteins”[6]. UCHL1 is expressed specifically in neurons and testes or ovaries [7]. In mouse testes, UCHL1 is expressed in gonocytes, already at 14 days of gestation [8]. Postnatally, between 8th and 16^th^ day, UCHL1 is localized in the spermatogonia, but in mature mice it appeared in spermatogonia, and in Sertoli cells [9].

Undescended testes exposed to higher body temperature, show degeneration of germ cells which occurs via apoptosis [10]. It is already known, that apoptosis of germ cells in undescended testes involves many genes including Bcl-2 proteins, p53 and caspases [6]. The ubiquitin-proteasome system regulate p53, caspase and Bcl-2 family proteins, and is crucial for the degradation of the defective germ cells in testes [6,11]. According to recent theories “ubiquitination in the epididymis may trigger apoptotic mechanisms that recognize and eliminate abnormal spermatozoa” and control the sperm quality [12].

In animal study, Du et al. showed that in undescended testes, UCHL1 concentrated into spermatocytes with apoptotic appearance to response to hyperthermia [13]. It is hypothesized, that UCHL1 accumulates in the spermatocytes due to the blockade of degradation or the up-regulation of its expression. The proof of this theory, can be found in animal study, in which spermatogenesis in UCHL1 deficient gad mice was resistant to apoptotic stress caused by heat [10].

To confirm findings from animal models, we wanted to evaluate the concentration of UCHL1 in the blood plasma of boys with cryptorchidism and if there is any correlation with patient age. To our knowledge this is the first human study assessing UCHL1 in connection with undescended testes.

To investigate the ubiquitin carboxyl-terminal hydrolase 1 (UCHL1) in blood plasma of boys with cryptorchidism, we used a novel technique Surface PLASMON RESONANCE Imaging (SPRI). So far several SPRI biosensors were used for clinical research to determinate e.g. 20S proteasome, matrix metalloproteinase-2, ubiquitin carboxyl-terminal hydrolase L1 (UCHL1), and immunoproteasome [14,15,16].

## Material and methods

### Patients

The study population comprised 50 boys aged 1–4 years (median = 2,4y.) with unilateral cryptorchidism. Exclusion criteria were: previous human chorionic gonadotropin treatment, an abnormal karyotype, endocrine or immunological disorders or any long-term medication. All of them were operated on and underwent successful orchidopexy. During the operation, testes were measured, and their the position and morphology evaluated.

### Control group

The control group represented 50 healthy, age matched boys (aged 1–4 years, median = 2,1y.), admitted to the Pediatric Surgery Department for planned herniotomy. All boys in the control group had their testes in the scrotum. They also underwent karyotyping before their surgical treatment.

The study was approved by the Ethics Committee of Medical University of Bialystok Poland. All parents gave their informed, written consent for the inclusion to the study, clinical and biochemical follow-up and for surgical treatment.

## Methods

Blood samples were drawn from the antecubital vein. Blood was collected in tubes containing EDTA. The serum was separated from cells by centrifugation. Serum samples were made anonymous and stored at –20oC.

### Procedure of UCHL1 determination

The ubiquitin carboxy-terminal hydrolase L1 (human recombinant UCH-L1, R&D System. Inc.) concentration was determined using the SPRI (Surface Plasmon Resonance Imaging) biosensor as described in the previous paper [18]. Gold chips were manufactured as described in other papers [17,18]. The gold surface of the chip was covered with photopolymer and hydrophobic paint.

According to Sankiewicz et al.: “Chips were rinsed with ethanol and water and dried under a stream of nitrogen. They were then immersed in 20mM of cysteamine ethanolic solutions for 2h and after rinsing with ethanol and water dried again under a stream of nitrogen. The rabbit monoclonal IgG2A antibody specific for human UCHL (R&D System. Inc.) were immobilized on the thiol monolayer under the suitable conditions. The antibody solution in a PBS buffer was activated with NHS (250 mM) and EDC (250 mM). Activation of the antibody was done by adding the mixture of NHS and EDC (1:1) in a carbonate buffer solution (pH 8.5) into the antibody solution and with vigorous stirring for 5 min at the room temperature. 3μl of this solution was placed on the active places with the amine-modified surface, and incubated at 37oC for 1h” [18].

“After this time the biosensor were rinsed with water. Next, serum samples (10 x diluted) were placed directly on the prepared biosensor. The volume of the sample applied on each measuring field was 3 μl. Time of the interaction with antibody was max 10 minutes. The biosensor was washed with water and HBS-ES buffer solution pH = 7.4 (0.01 M 4-(2-hydroxyethyl) piperazine-1-ethanesulfonic acid, 0.15 M sodium chloride, 0.005% Tween 20, 3 mM EDTA), BIOMED, Lublin, Poland) to remove unbound molecules from the surface” [18].

SPRI measurements for protein array were performed as described in the previous papers:”The SPRI signal was measured at a fixed SPR angle on the basis of registered images. The first image after immobilization of the antibody was taken. Then, the second image after interaction with UCH-L1 was taken. The SPRI signal was obtained by subtraction of the signal after and before interaction with a biomolecule, for each spot separately. The contrast values obtained for all pixels across a particular sample single spot were integrated. Then, the SPRI signal was integrated over the spot area. NIH Image J version 1.32 software was used to evaluate the SPRI images in 2D form and to convert of numerical signal to a quantitative signal (A.U.)” [17,18].

The results were assessed using a linear section of the calibration graph (0.1–2.5 ng/ml, R2 = 0.9926) after an adequate sample dilution with PBS buffer. The curve was made just before the assessment. The precision of measurements was approximately 10%.The levels of INSL-3, AMH and inhibin B were assessed using commercial enzyme-linked immunosorbent assay ELISA kits Beckman Coulter and Uscn Life Science Inc.

## Statistical analysis

Statistical analysis was performed using the STATISTICA PL release 12.5 Program. All the results are presented as median with 25th and 75th percentiles. Because tested parameters in plasma of patients did not pass the normality Shapiro-Wilk test continuous variables were compared by the nonparametric Mann-Whitney U test (two-sided nature)between study groups. Correlations were studied by using the Spearman Correlation Test. A p value of p<0.05 indicated a statistically significant difference.

**Fig 1:**
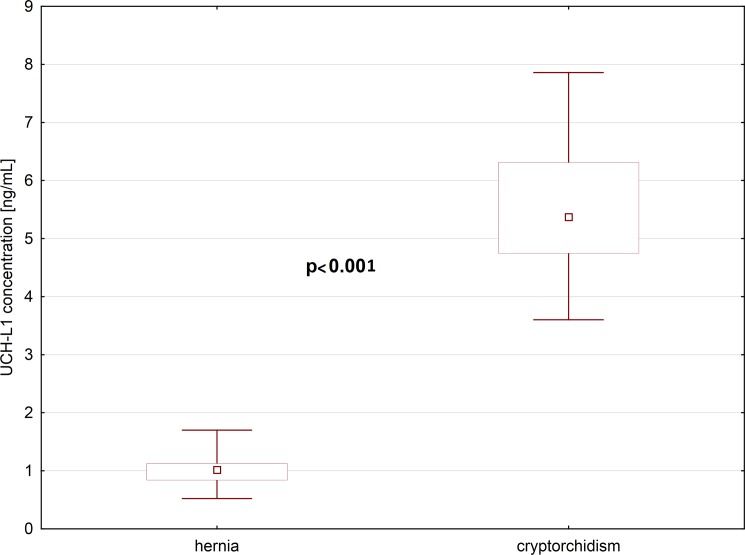
Plasma UCHL-1 concentration in children with hernia and cryptorchidism (N = 50).

**Fig 2:**
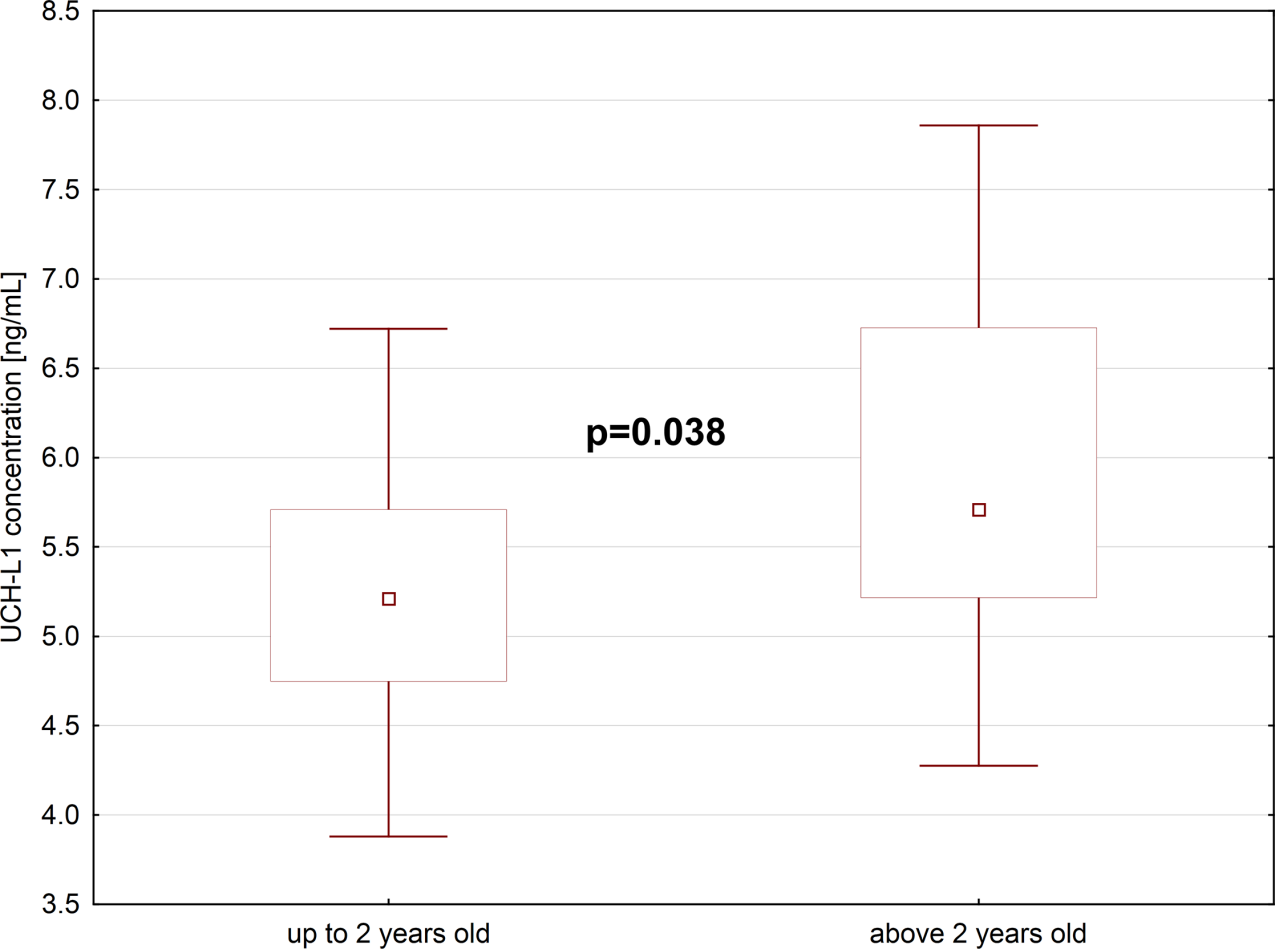
Plasma UCHL-1 concentration in children with cryptorchidism of age up to (N = 20) and above 2 years old (N = 30).

There was no statistically significant difference in the age distribution of the group of boys with cryptorchidism and boys with inguinal hernia, who served as controls. All boys had karyotypes 46XY. In most cases of cryptorchidism, the undescended testes were found in the superficial inguinal pouch (n = 46), but in two boys were located in the external ring of inguinal canal, and also two patients had their testicles located in the abdomen. Generally, undescended testes were smaller in comparison to the testes positioned normally (mean 1cm and mean 1,5cm respectively). The median concentration of UCHL1 in the blood plasma of boys with cryptorchidism, was 5-folds higher than in boys with inguinal hernia, whose testicles were located in the scrotum (Fig 1, S1 Fig). The difference was statistically significant (p<0.001). We also noticed statistically significant difference between UCHL1 levels in boys with cryptorchidism up to 2 years old, and above 2 years old. Older boys, whose testicles since birth were located in the inguinal pouch or in the abdominal cavity, had higher concentration of UCHL1 in their blood plasma, than boys from younger group. The difference was statistically significant (p = 0.038) (Fig 2). In the group of cryptorchid boys, we also found slightly lower concentrations of INSL3, without statistical significance, and without correlation with UCHL1 levels. We did not find statistically significant differences in the levels of AMH and inhibin B in the group of boys with cryptorchidism and boys with inguinal hernia, and there were no correlations between UCHL1 and AMH or UCHL1 and inhibin B in those groups (Table 1).

**Table 1:** The statistic parameters of UCH-L1, INSL3, AMH and inhibin B concentrations in plasma of children diagnosed with hernia and cryptorchidism.

	Cryptorchid n = 50	Control group n = 50	P value
UCHL1 ng/ml	5.83 (±2.02)	1.04 (±0.33)	<0.05
INSL3 pg/ml	2672.000 (±1334.4)	3267.500 (±1505.9)	= 0.05
AMH ng/ml	118.200 (± 59.996)	111.500 (±59.5)	>0.05
Inhibin B pg/ml	113.770 (± 54.241)	129.575 (±78.646)	>0.05

* A p value <0.05 is considered to show a significant difference between groups

## Discussion

Sperm production in the testis depends on equilibrium between division and loss of germ cells. During spermatogenesis, apoptosis limits the quantity of germ cells and eliminates those which carry DNA mutations, occurring through chromosomal crossing over during the first meiotic division [19]. Kwon et al. indicate that ubiquitination targets proteins for degradation, and by this, takes part in the control of sperm quality during sperm maturation [10]. UCHL1 is expressed at high levels in both testis and epididymis, and defective spermatozoa and proteins in these cells become ubiquitinated during transit through the epididymis [6,10,19]. According to Wang et al. “overexpression of UCHL1 affects spermatogenesis during meiosis and, induces apoptosis in primary spermatocytes” [20]. Contrary to that, Kwon et al. reported that in mice, UCH-L1 can be detected mainly in spermatogonia and somatic Sertoli cells, so it might function in mitotic proliferation in spermatogenesis [6,10,19]. Still, the spermatocytes are the main type of germ cells undergoing apoptosis in cryptorchidism [21].

Apoptosis of germ cells caused by elevated temperature in undescended testes, involves many determinants, such as Bcl-2 famliy proteins, the tumor suppressor protein p53, and caspases [22,23]. P53 protein is highly expressed in the testis, increase of its level caused by high temperature, results in intensified germ cell apoptosis of germ cells and less production of spermatozoa [24]. In addition Bcl-2 family and IAP (inhibitor of apoptosis protein) family are other important factors regulating apoptosis, and together with caspase members, are targets for ubiquitination [25]. UCHL1 occurred abnormally in the spermatocytes in response to abdominal hyperthermia, and in association with Jab1 and p27kipl [13]. Under the physiological conditions, UCHL1, Jab1 and p27kipl were not expressed in spermatocytes, Du et al found that cyclin-dependent kinase inhibitor p27kipl increased in some spermatocytes, just paralleled to Jab1 and UCHL1, in response to heat stress, and apparently concentrated in nuclei of apoptotic spermatocytes [13]. Probably p27kipl mediate the UCHL1-involved apoptosis directly through its abnormal increase in spermatocytes in response to heat-stress [13]. All this findings suggest apoptotic cell death is regulated mainly by ubiquitination.

In human males, spermatogenesis begins at puberty in seminiferous tubules in the testicles and go on continuously [1]. In prepubertal boys, immature germ cells–spermatogonia, proliferate continuously by mitotic divisions around the outer edge of the seminiferous tubules, next to the basal lamina [1].

In boys with cryptorchidism, according to Chung: “The reduction in germ cell count starts as early as 6 months of age and is dependent on the position of the testis” [1]. Study by Wilkerson et al. who compared fertility potential of undescended testes, by age groups in children revealed, that “the higher the testicular position at the time of treatment, the fewer the number of germ cells” [26] According to human studies “spermatogenic index decreases significantly by 9 months of age”‘ so operation of undescended testes at this age or before, may stop testicular degeneration and improve chances for future fertility [26]. Moreover, Tasian et al. reported “a significant 2% risk per month of severe germ cell loss and 1% risk per month Leydig cell depletion for each month a testis remains undescended” [27]. According to the same research “the odds of germ cell loss almost double for each age range at the time of orchidopexy” [27].

UCHL1 was first identified in the brain and testes, and accounts for 1–2% of brain soluble proteins [28,29]. Studies on adults demonstrated that increased concentrations of UCHL1 can be found in serum after traumatic brain injury [28,29]. Also in children after head trauma, increased serum levels of UCHL1 correlated with worse outcome [30]. In control groups, levels of UCH-L1 were significantly higher in the first year of life, than those measured in older children, the highest levels found below 3 months of age. Most probably the cause of that is underdevelopment of the blood–brain barrier in infants. Contrary to this observation, according to Ferguson et al. serum levels of UCH-L1 increase with age in young healthy adults [31].

Heat induced alterations in tight-junction proteins in undescended testis proteins cause higher permeability of blood-testis barrier [32]. We speculate, that it can be the source of UCHL1 in blood plasma originating from the apoptotic germ cells.

We believe, that the results of our study, indicate that Uchl1 concentrations in the blood plasma of boys with cryptorchidism, reflect the heat-induced apoptosis of germ cells. The levels of UCHL1 in the blood plasma of our patients with cryptorchidism, were 5-folds higher than in controls, whose testicles were located in the scrotum. Furthermore, we also noticed that UCHL1 concentrations differed between the group of boys with cryptorchidism up to 2 years old, and above 2 years old. Older boys, whose testicles since birth were located in the inguinal pouch or in the abdominal cavity, had higher concentration of UCHL1 in their blood plasma, than boys from the younger group. This observation exclude potential bias of serum concentrations of UCHL1 correlating with age in young children, as according to Berger et al. “UCH-L1 concentrations during the first year of age were significantly higher than those measured at later ages” [30]. In the group of cryptorchid boys, we also found slightly lower concentrations of INSL3, without statistical significance and without correlation with UCHL1 levels. INSL3 is expressed in Leydig cells in testis, shortly after the onset of testicular development, and controls the thickening of the gubernaculum anchoring the testis to the inguinal region. Kumagai et al. in animal model showed that “deficiency of INSL3 resulted in cryptorchidism or defects in testis descent due to abnormal gubernaculum development” [33]. We did not find statistically significant differences in the levels of AMH and inhibin B in the group of boys with cryptorchidism and boys with inguinal hernia and it also did not correlate with UCHL1 concentration. AMH is secreted by immature Sertoli cells during the 8th week of gestation, and is responsible for the regression of Mullerian ducts in the male fetus [34]. Mutations in the the AMH receptor cause the persistent Mullerian duct syndrome in males, and disrupts the descend of the testis [34]. Surprisingly in this group of patients, the AMH levels did not differ significantly between cryptorchid boys and boys with inguinal hernia, contrary to the results of previous studies [34]. As to Inhibin B, it is produced by the Sertoli cells in the prepubertal testis, and controls FSH secretion by a negative feedback mechanism [34]. Before puberty levels of inhibin B “reflect the presence of Sertoli cells, and not the germ cells” [34]. We did not assessed the testosterone levels, although an infant boy may have slightly elevated its levels, but at the age of six months it will drop to a range of undetectable to 20 ng per dL of blood until a boy reaches puberty [35].

Most probably, higher UCHL1 concentrations in older boys with undescended testicles, express intensity of apoptosis of germ cells, more extensive when testicles are subjected to heat-stress for longer period.

The aetiology of cryptorchidism is multifactorial, genetic, hormonal and environmental factors playing a role [36–40]. We believe, that our study may help to understand its molecular consequences, influencing future fertility in patients with undescended testes.Uchl1 concentrations in the blood plasma of boys with cryptorchidism, may reflect the heat-induced apoptosis of germ cells. Higher UCHL1 concentrations in older boys with undescended testicles, probably express intensity of germ cell apoptosis, more extensive when testicles are subjected to heat-stress for longer period. Further analyses of UCHL1 may help to elucidate its role in mechanisms influencing spermatogenesis.

## Supporting information

S1 Fig Blood plasma levels of UCHl1 in boys with cryptorchidism and inguinal hernia. (XLSX)
